# The Use of Mind-body Medicine in Chronic Pain Management: Differential Trends and Session-by-Session Changes in Anxiety

**Published:** 2016-03-30

**Authors:** David Cosio, Sujata Swaroop

**Affiliations:** 1 Anesthesiology/Pain Clinic, Jesse Brown VA Medical Center, USA; 2 Pain Clinic/Psychology, Jesse Brown VA Medical Center, USA

**Keywords:** Acceptance and commitment therapy, Cognitive-behavioral therapy, Chronic pain, Trend analysis, Brief therapy, Beck anxiety inventory

## Abstract

The evidence to date suggests that the use of mind-body medicine in chronic pain management can improve physical and psychological symptoms. However, past research evidence has largely relied on global measures of distress at pre- and post-intervention. Even though it is plausible that reported anxiety occurs in the context of pain, there is also evidence to suggest a reciprocal relationship. Thus, the purpose of the current study was to determine the differential impact that mind-body medical interventions have on anxiety among Veterans with chronic, non-cancer pain. The current study utilized multiple, repeated assessments of anxiety to better understand changes made over time between two mind-body interventions (Acceptance and Commitment Therapy (ACT) and Cognitive Behavioral Therapy (CBT)) used for chronic pain management. Ninety-six Veterans elected to participate in either intervention following the completion of a pain health education program at a Midwestern VA Medical Center between November 3, 2009–November 4, 2010. A 2 × 7 repeated measures multivariate analyses of variance indicated significantly lower levels of global distress by the end of both the ACT and CBT interventions. Trend analysis revealed differential patterns of change in levels of anxiety over time. Helmert contrast analyses found several modules of ACT were statistically different than the overall mean of previous sessions. Implications related to timing and patterns of change for the interventions are discussed.

## Introduction

A positive relationship exists between pain and anxiety in clinical settings. Past research has found these associations to be larger than those between pain and depression [1]. In fact, a recent study found that relief from anxiety and low baseline depression were the most important predictors for pain relief and the most strongly associated with functional improvement [2]. Past research has also shown that the strongest associations between pain and anxiety were observed with panic and post-traumatic stress disorders as defined by the DSM-IVR [3]. These findings suggest there needs to be an improved effort regarding the detection and treatment of anxiety disorders in pain treatment settings. The human experience of pain is not merely somatic, but it also varies according to mental, emotional, and physical factors that work via similar mechanisms and have synergistic effects [4]. For any two conditions, there are several possible relationship scenarios. Even though it is plausible that reported anxiety occurs in the context of pain, there is also evidence to suggest a reciprocal relationship [5]. The mutual maintenance model holds that anxiety maintains or exacerbates symptoms of pain, and vice versa [4,6]. Anxiety levels have been shown to predict pain severity and behaviour in patients who suffer from chronic pain [7]. Certain signs of anxiety, such as catastrophizing and hypervigilance, have been shown to play key roles in chronic pain. Thus, comorbid anxiety has implications for the impact and outcome of chronic pain [5].

Mind-body medicine emphasize in engaging both in mind and body to promote stress reduction and well-being by changing the manner in which patients respond to their stressors. Any intervention that changes a patient’s mental or emotional state will produce corresponding changes in the body and could therefore be called a “mind-body” intervention [1]. Thus, mind-body therapies can also be used to treat and/or prevent a variety of conditions, including chronic pain disorders [8]. Some of the most commonly used therapies include mindfulness, as practiced in Acceptance and Commitment Therapy (ACT), and relaxation techniques, as trained in traditional Cognitive-Behavioural Therapy (CBT). Both ACT [9–13] and CBT [14–21] have been recognized in past literature as empirically supported treatments for chronic pain. In addition, research has provided evidence that ACT compares favourably with traditional CBT in the treatment of chronic pain [22–25]. Recently there has been some debate about utilizing global measures of distress as pre- and post-assessments for chronic pain treatment, given its multiple dimensions [26,27]. Unfortunately, current literature lacks consensus for guiding clinicians regarding selection of these measures. For example, the Beck Anxiety Inventory^®^ (BAI) [28] has been identified as a measure of pain-related anxiety [29], but other scholars warn there may be potential for misinterpretation of anxiety symptoms as symptoms of pain [30]. However, utilizing this measure for multiple, repeated assessments of anxiety may serve as corroboration to pre- and post-assessments when measuring distress [31]. Furthermore, assessing change session-by-session may allow future modifications to be made to enhance results, give therapists a better understanding of effective techniques, and provide evidence of treatment effectiveness to stakeholders [32].

**Figure 1:**
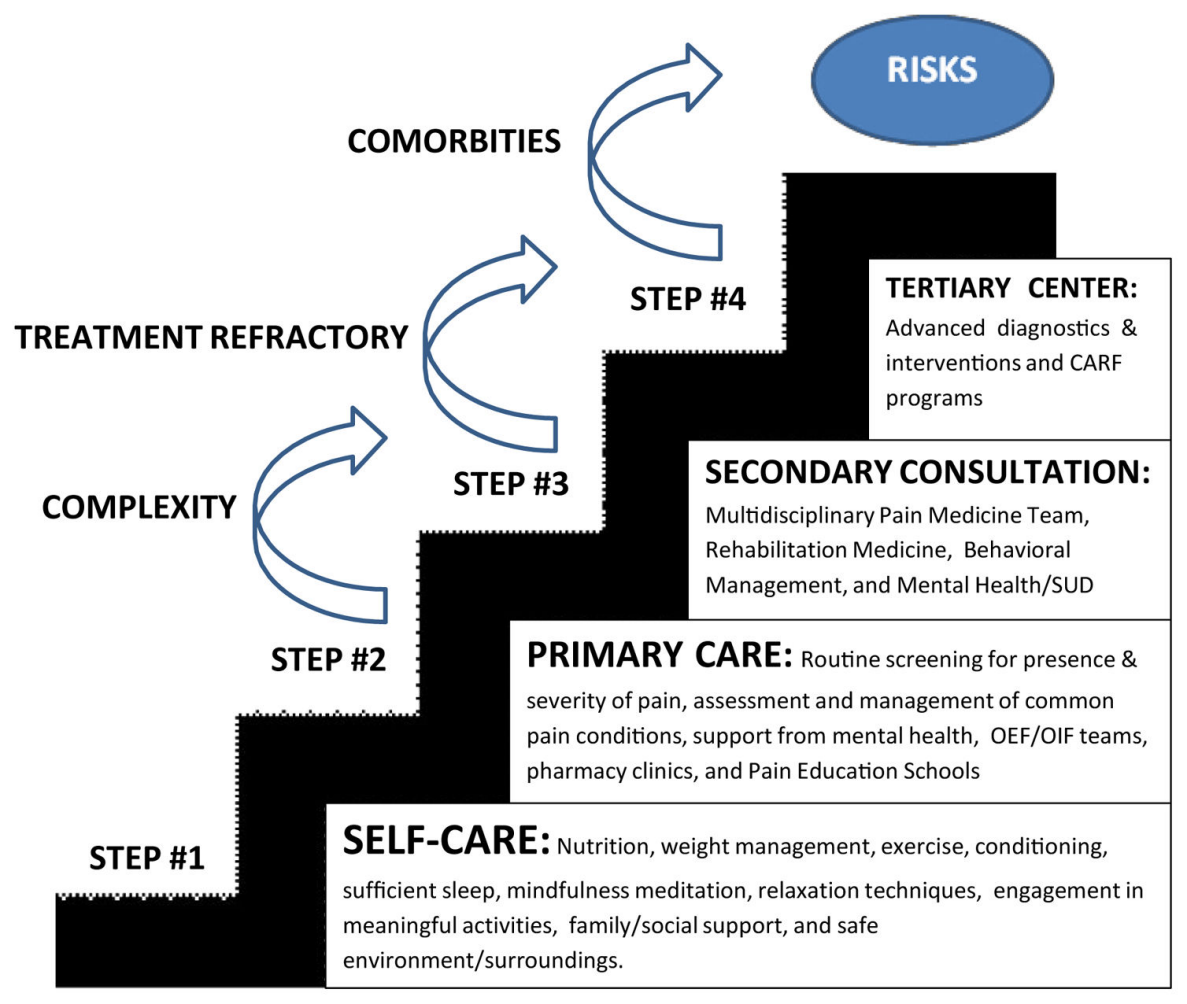
The VA’s stepped care model of pain management.

Pain is one of the most common reasons Veterans consult with their primary care providers [33]. In fact, about half of the Veterans within the VA experience pain regularly [34]. Veterans with chronic pain are often more complex than civilians due to difficulties returning to their private life and the influence of their past military service on their pain [35]. Previous research has found that past military service may contribute to hypersensitivity [36–38], and may serve as an exemplar population to determine the efficacy of mind-body medical interventions for the treatment of physiological and psychological distress. In 2009, the VA advocated for the “Stepped Care Model of Pain Management” as a best practice model [39]. The stepped care model gives providers the ability to escalate treatment options to include specialized care and multidisciplinary approaches (Figure 1). The purpose of the current study was to determine the differential impact that mind-body medical interventions have on psychological distress among Veterans with chronic, non-cancer pain within this framework. To this end, change in distress across the two treatment conditions was measured using multiple assessments of anxiety over many weeks, allowing the examination of different patterns over the course of treatment.

## Materials and Methods

### Participants

Two hundred and six Veterans were introduced to the mind-body medical interventions while participating in a pain health education program during Step #2 of the care model [40]. One hundred and ten Veterans elected to participate in either intervention following the completion of the pain health education program at a Midwestern VA Medical Center between November 3, 2009–November 4, 2010. The current study had no exclusion criteria in order to reflect the population as it is encountered in clinical practice.

### Procedure

Potential candidates were then scheduled for the next available selected intervention. Groups have been shown to be useful in breaking down isolation, enhancing motivation, and providing the benefits of learning from the insights and experiences of other group members. In addition, a recent literature review of various aspects of delivering psychosocial interventions for chronic pain found that group-delivered courses showed more beneficial effects [41]. Thus, each Veteran was subsequently scheduled for 10-weeks of one-hour, ACT or brief CBT group sessions. Enrollment in each group was capped at 15 Veterans per cohort. Participants were seen on a weekly or biweekly basis depending if there was a holiday scheduled. Therapists in this study were licensed clinical psychologists coupled with advanced graduate trainee therapists who had been trained to work with Veterans. Prior research has supported the use of graduate trainees in mind-body medical interventions [42]. Veterans voluntarily participated in the program and were free to withdraw at any time. Veterans were given free parking validation/transportation reimbursement if they were in attendance and were qualified for such programs. The current study’s protocol was reviewed and approved by the affiliated university’s Institutional Review Board and the VA’s Research and Development office. A wavier of informed consent was granted due to the retrospective nature of the study and the minimal risk to subjects who participated.

### Mind-body interventions

#### Cognitive-behavioral therapy (CBT)

CBT is a structured, time-limited, present-focused approach to psychotherapy that helps patients develop strategies to modify dysfunctional thinking patterns and maladaptive behaviors in order to assist them in resolving current problems. The 10-week CBT treatment group was a brief protocol based on an empirically supported, “Treatments That Work” manual [43], and sessions addressed different ways to cope with and reduce experience of chronic pain (Table 1). The protocol reviews pain education topics, introduces cognitive concepts, and teaches behavioural strategies. CBT therapy for chronic pain has been disseminated nationwide by the VA Office of Mental Health Services and National Pain Management Program Office.

#### Acceptance and commitment therapy (ACT)

ACT is distinct from other mindfulness-based interventions, such as Mindfulness-Based Stress Reduction (MBSR), Mindfulness-Based Cognitive Therapy (MBCT), and Dialectical behaviour therapy (DBT). ACT is a form of clinical behaviour analysis employed in psychotherapy that uses acceptance and mindfulness strategies mixed with commitment and behaviour-change strategies to increase psychological flexibility. MBSR uses a combination of mindfulness meditation, body awareness, and yoga to help people become more mindful. MBCT uses traditional CBT methods and adds in mindfulness meditation. Mindfulness is a “core” exercise used in DBT, which combines standard cognitive behavioural techniques for emotion regulation with concepts of distress tolerance derived from Buddhist meditative practice. The 10-week ACT treatment group followed an amalgamation of established protocols [44,45] and a self-help workbook [46] in which sessions addressed participants’ relationship with their thoughts, feelings, memories, and bodily reactions to pain. The current amalgamated protocol was established and served as a best course of treatment within the ACT model for the chronic pain population (Table 1).

### Assessment instruments

As part of the introduction and conclusion of each intervention, Veterans completed a pre- and post-intervention assessment which included the Numeric Rating Scale (NRS-11) [47] and the Brief Symptom Inventory^®^-18 (BSI-18) [48]. Veterans also completed the Beck Anxiety Inventory^®^ (BAI) [28] before the beginning of each group session in both interventions.

#### Numeric rating scale (NRS-11)

The NRS-11 is an 11-point scale for patient self-reporting of pain for adults and children over 10 years old. Scores range from 0 to 10, with “0” meaning no pain, “1 to 3” mild pain, “4 to 6” moderate pain, and “7 to 10” severe pain.

#### Brief symptom inventory^®^-18

The BSI-18 is an 18-item measure of general psychological distress used with adult medical and community populations. The BSI-18 subjectively measures the participants’ degree of global distress. Scores are calculated from responses indicated on a five-point Likert scale. This measure has been shown to have satisfactory reliability for the measure of global distress [48]. The internal consistency for the BSI-18 in the current study was good (α = 0.84). Permission to use the BSI-18 is inherent in the qualified purchase of the test materials.

#### Beck anxiety inventory^®^

The BAI is a 21-item measure of anxiety severity used extensively with adults. The BAI subjectively measures how the participant has been feeling in the last week, with each item representing one symptom of anxiety. The BAI functions more adequately in anxiety disorders with a high somatic component, such as panic disorder, and has been shown to be less contaminated by depressive content. A total anxiety score is calculated from responses indicated on a four-point Likert scale. The BAI was chosen based on its utilization to measure anxiety by past pain management investigators [26,49]. The BAI is the third most utilized research measure of anxiety [50]. The BAI has demonstrated high internal consistency, with Cronbach’s alphas 0.90–0.92, and satisfactory test-retest reliability, *r* = 0.75 [28,49]. The internal consistency of the BAI in the current study was good (α = 0.91). Permission to use the BAI is inherent in the qualified purchase of the test materials.

### Data analyses

A one-way analysis of variance and independent samples t-tests identified differences on demographic and outcome variables at baseline. The relationship between the BAI and the BSI-18 was measured using Spearman’s correlation. The 21 items of the BAI were then subjected to principal components analysis (PCA) followed by a varimax rotation with Kaiser normalization using the SPSS 20 to cross validate the measure’s factor structure. A last-observation-carried-backward approach [51] was used for missing data on items on the pre-assessment and a last-observation-carried-forward approach [52] was used for missing data on items on the post-assessment. The Power and Sample Size Program [53] was utilized to calculate sample size using an anticipated effect size (Cohen’s d) of 0.50, a desired statistical power level greater than or equal to 0.80, and a probability level less than or equal to 0.05. The minimum total sample size (pairs of subject scores) was N = 33.

The primary intervention outcome analysis was a 2 × 7 repeated measures (RM) multivariate analyses of variance (MANOVA). The ACT and brief CBT protocols were defined as “Intervention Condition” which served as the between-subjects factor and several weekly assessment points (session #2- session #8) were defined as “Time” and served as the within-subjects factor. An efficacy subset analysis strategy was utilized. A trend analysis was then computed to explore the presence and nature of the relationship between “Intervention Condition” and “Time” employing polynomial functions. Furthermore, Helmert contrast analyses were conducted in order to test how each module added to the trend over time.

## Results

### Participant characteristics

**Figure 2:**
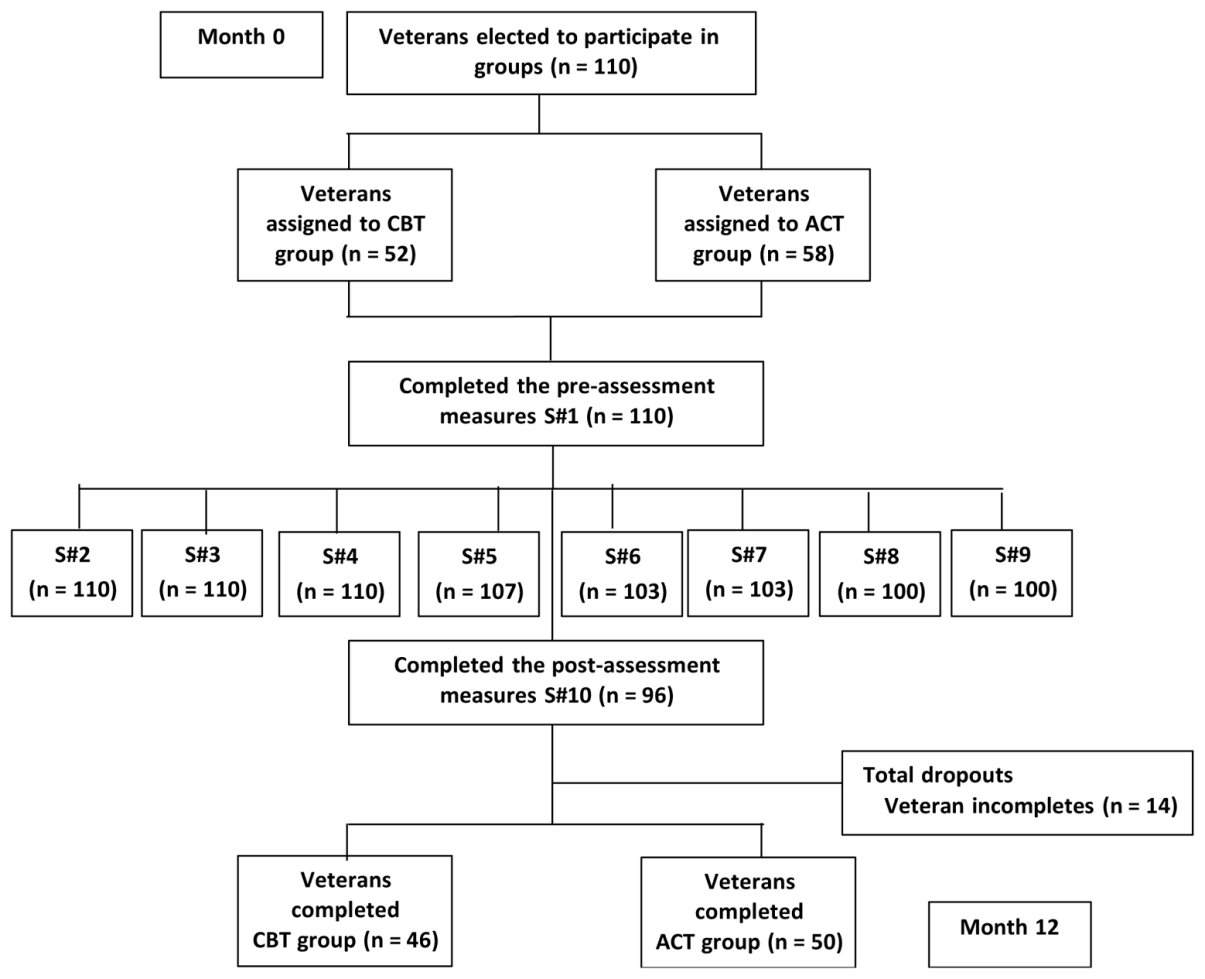
Flowchart of study participants.

Eighty-seven percent (N = 96) of the sample elected to complete the pre- and post-intervention assessments, and only their responses were included in the current study (Figure 2). Veterans had mixed idiopathic chronic, non-cancer pain conditions. Most Veterans were African American (78%), but 16% were Caucasian and 6% identified as being Hispanic/Latino. Most were males (90%), but there were also a representative sample of female Veterans (10%). Most of the Veterans were 45 to 54 years old (40%) or 55 to 64 years old (38%), and the youngest returning Veterans (17–24 years old) were not a represented age group. The reported pain score at baseline for all the Veterans in the current study was “6.02;” at post-intervention the average pain score remained similar “5.82” (moderate pain). The mean for the baseline BSI-18 global distress score was 25.63 (SD = 13.06), and the BAI baseline total score was 19.50 (SD = 11.23), which indicate a moderate level of psychological distress among the Veteran sample. The average ACT group member attended 7 out of 10 sessions (SD = 1.86), while the average brief CBT group member attended 8 out of 10 sessions (SD = 2.03). Five, group offerings of the ACT protocol and four of the brief CBT protocol were conducted during the time of the investigation.

### Differences at baseline

Independent samples t-tests revealed there was no significant differences between the mind-body medical interventions in reference to members’ sex demographic (*p* = 0.24). ANOVA findings indicated there was no differences in group assignment (*p* = 0.10) or race/ethnic demographics (*p* = 0.71) between the interventions. ANOVA also revealed there was no significant difference between the interventions on baseline BSI-18 scores, F(1,94) = 3.12, *p* = 0.08, and the baseline BAI total scores, F(1,94) = 1.04, *p* = 0.31.

### Psychometric outcomes

The Spearman correlation between the BAI and BSI-18 was *r_s_* = 0.75, *p* = 0.00. These findings build upon previous research validating the utility of the BSI-18 [48] and the BAI [28,49,54] as measures of psychological distress among different populations. Prior to performing PCA, the suitability of data for factor analysis was assessed in accordance with previous recommendations [55]. The Kaiser-Meyer-Oklin value [56,57] was 0.85 and the Barlett’s Test of Sphericity [58] reached statistical significance *p* = 0.00. PCA revealed the presence of five components with eigenvalues exceeding Kaiser’s criterion (Table 2). Only one component had an eigenvalue exceeding the corresponding criterion value for a randomly generated data matrix of the same size [59]. Thus, the current findings support past research which has used the BAI as a unidimensional measure of distress in a pain population [49].

### Intervention outcomes

A 2 × 7 RM MANOVA found a significant multivariate “Intervention Condition × Time” interaction, F(6, 87) = 2.18, *p* = 0.05, Wilk’s Lambda = 0.87, and a significant main effect for “Time” on the combined set of measures, F(6,87) = 2.34, *p* = 0.04, Wilk’s Lambda = 0.86. There was no significant main effect for “Intervention,” Wilks’ λ = 0.95, F(6,87) = 0.63, p = 0.73, which indicates that ACT and brief CBT were not significantly different on their impact on the dependent measures of pain severity and global distress. A significant main effect for time was not obtained for the primary measure of pain severity, F(1,94) = 1.85, *p* = 0.18. The Mauchly’s Sphericity Test result was 0.05 (*p* = 0.00).

Polynomial functions indicated that the linear (*p* = 0.04) component was significant for “Intervention Condition × Time.” Findings also showed that the quadratic (*p* = 0.05) component was significant for “Time.” In accordance with past research, a significant effect meant that the associated line fit the means at better than chance levels [60]. The trend analysis revealed that the groups show different linear trends over time. There was not a significant treatment effect for “Intervention Condition,” F(1,92) = 0.01, *p* = 0.92.

**Figure 3:**
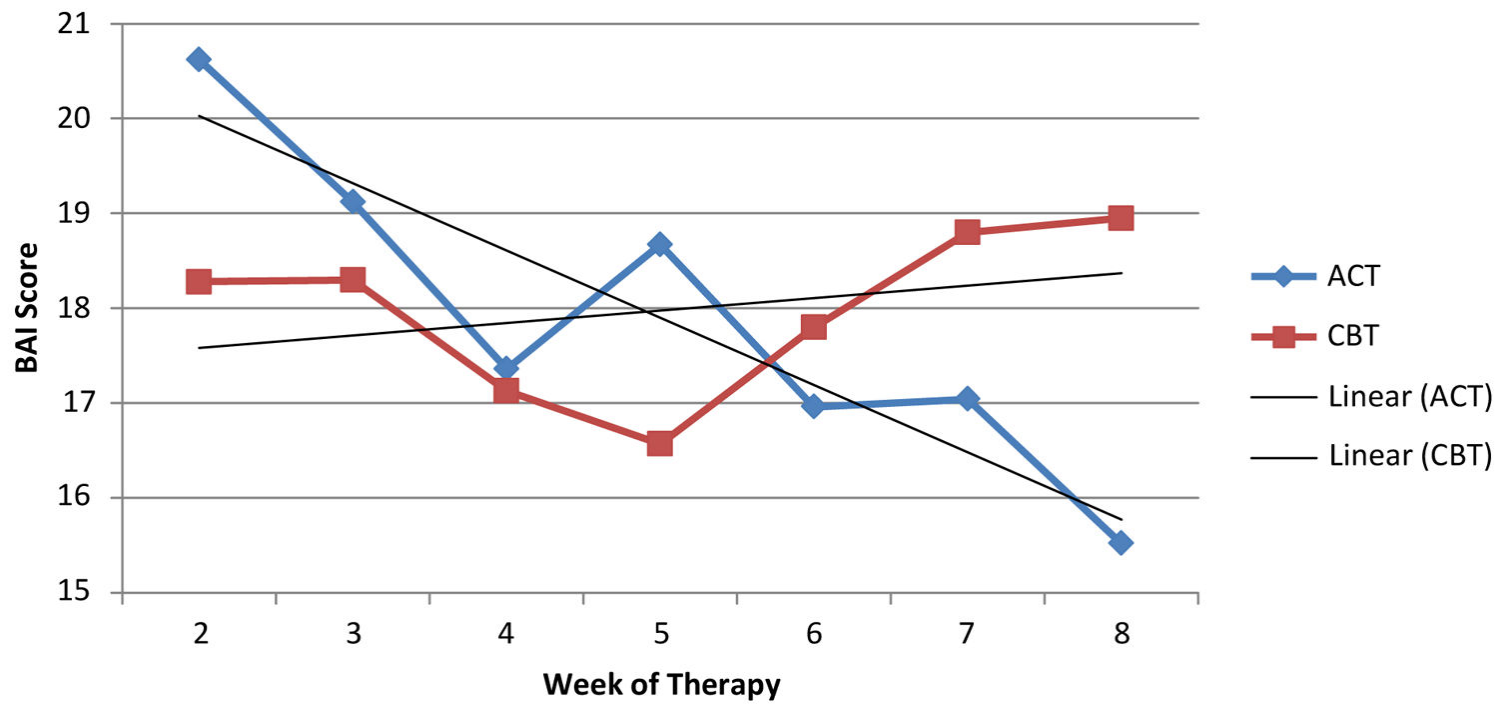
Screeplot for polynomial functions.

Helmert contrast analyses did find that the means of several modules of the ACT protocol were statistically different than the overall mean of the previous modules. However, the analyses did not find that the means of any module of the brief CBT protocol were statistically different than the overall mean of the previous modules (Figure 3).

## Discussion

While prevailing literature clearly demonstrates the efficacy of mind-body medical interventions for the treatment of psychological and physiological distress, research has traditionally focused on changes in symptoms from pre- to post-treatment [27]. Although establishing efficacy is vital, the bias toward efficacy in extant research has led to the neglect of important questions regarding whether mind-body medical interventions work because of the mechanisms specified by theory [61]. Some researchers have proposed measuring change across therapies in order to improve treatment, to enhance clinical science, and to provide accountability. The current study examined which elements of mind-body medical interventions are contributing to decreased psychological and, in turn, physiological distress, and when change is occurring by measuring change session-by-session in a pain treatment setting. The current study did not find any significant difference over time in pain severity in either mind-body medical intervention. These findings are inconsistent with past research [10,12,13,25]. Perhaps, Veterans who suffer from chronic pain may be a unique group of individuals due to the dualism of active duty and civilian life [25]. These compounded experiences may maintain pain at a moderate level (“4–6” on NRS-11). The current Veteran sample reported moderate pain scores at pre-and post-assessment, which supports this notion. Furthermore, the permanence of chronic pain may undermine recovery and remove a sense of hope or optimism. Therefore, a better adjustment to continuing pain may prove to be a more realistic goal. Thus, further exploration of psychological distress was warranted.

More specifically, this study found a linear trend for Veterans engaged in the ACT intervention group, reflecting a steady decrease in reported distress through sessions focused on learning about the relationship between psychological inflexibility and pain maintenance; letting go of control; identifying one’s values; and learning about cognitive defusion. This could be an illustration of a rapid early response. However, after session #4, distress patterns rose slightly and did not contribute to a statistical difference when compared to previous modules. The progress then continued to decrease through sessions focused on mindfulness; reaching acceptance using self-as-context; and making a commitment to action. In contrast, the current study found a quadratic trend for Veterans engaged in the CBT intervention group, reflecting non-significant decreases in reported distress through sessions focused on diaphragmatic breathing; progressive muscle relaxation and guided imagery; learning about cognitive errors and the ABC model; cognitive restructuring; and stress response/stress relief. Again, this could be an illustration of a rapid early response. However, after session #6, distress patterns increased and remained high through sessions focused on activity pacing and pleasant activity scheduling (Figure 3). Note, the mean distress score at session #8 for ACT was in the mild range (BAI = 15) but remained in the moderate range (BAI = 19) for the CBT group.

Further review of the polynomial screeplot (Figure 3) revealed that the scores on the BAI converged around session #6 for the two interventions before surging in different directions. Of note, it is at the beginning of session #6 that Veterans have completed the lesson on cognitive defusion in ACT and cognitive restructuring in brief CBT. ACT and brief CBT overlap to a large extent in shared techniques and strategies with respect to behavioural interventions, such as exposure exercises, problem solving skills, role playing, etc. The major distinction between the ACT and brief CBT interventions lies within their cognitive strategies, which may provide context for the different trends observed. Thus, the results of the current study are consistent with the notion that substantial changes in outcomes should occur following large changes in the assumed mechanism factors [27]. The core message of the ACT intervention is to teach individuals to defuse and distance themselves from their pain instead of suppressing internal experiences as taught in CBT [62]. Perhaps ACT had a more linear trend because cognitive defusion is less demanding of self-regulatory capacity and therefore able to augment an individual’s ability to engage in self-regulation [63]. Perchance, the brief CBT intervention had a more quadratic trend because cognitive restructuring is less adaptive and more cognitively demanding than appraising pain experiences [64]. Despite both ACT and brief CBT using manual-based, empirically supported treatment strategies, the findings of the current study suggests that ACT may be more time efficient than brief CBT.

Several limitations in the current study should be noted. First, Veterans self-selected into either the ACT or the brief CBT intervention group. There was also no control group; thus, treatment effects could not be unambiguously reported according to intervention group. However, pain experts have noted that strong outcomes in patients with long-term and intractable conditions together with a clear pattern of results from consequent assessment measures yields a high likelihood that such findings relate to the specific patterns within the respective intervention [65]. Secondly, a potential problem with the current study is its reliance on self-report measures, which may be subject to social desirability biases. However, utilizing the BAI as a frequent assessment did allow for direct measures conducted closely in time and in situation to the behaviour patterns of interest. According to pain scholars, this may reduce report bias and may reduce the possibility of method effects inflating observed relations [65,66]. Another consideration may be that some changes on the outcome measures were the result of participants engaging in other pain management modalities while in the current study and not from the mind-body medical interventions delineated. Finally, the self-regulation skills and abilities of all Veterans with chronic, non-cancer pain may differ as the current sample was predominately African American and did not include a representative sample of the youngest returning soldiers (18–34 years old) when compared to the typical Veteran profile [67].

## Conclusions

In summary, both mind-body medical interventions for chronic pain in the current study experienced a decrease in Veteran reported anxiety. Overall, the current study provided evidence of the treatment effectiveness of both interventions for chronic pain. Yet multiple assessments over several weeks of each intervention indicated distinctive patterns in such trends. Assessing change session-by-session per intervention suggested future modifications should be made to enhance results. For example, the current study suggests that ACT may be more time efficient then CBT for pain groups. Patients enjoy rapid treatment gains. Time efficiency can improve the credibility of the treatment, increase motivation for further change, and lead to increased cost-effectiveness which could make treatment accessible to more individuals in need of assistance. In addition, weekly assessments may have provided a better understanding of effective techniques. For example, each session of the ACT protocol seems to have contributed to a decrease in anxiety except the module immediately following the introduction of cognitive defusion. Despite the findings that ACT was able to enhance self-regulation more than brief CBT, the concept of cognitive defusion may still prove to be too cognitively demanding for patients with chronic pain and additional support or instruction around this concept may prove beneficial. The current examination of the patterns and timing of change per ACT and brief CBT groups, as measured by session-by-session administrations of the BAI, may assist in improved clinical services and encourage future research in chronic, non-cancer pain treatment among Veterans.

## Figures and Tables

**Table 1:** Comparison of the 10-week ACT and traditional CBT protocols.

S. No.	Cognitive-behavioral therapy protocol	Helmert contrast P-value	Acceptance and commitment therapy protocol	Helmert contrast p-value
1	Education on chronic pain	-	Introduction to ACT	-
2	Theories of pain and diaphragmatic breathing	-	Controlling your pain	-
3	Progressive muscle relaxation and visual imagery	0.96	What do you value?	0.01
4	Automatic thoughts and pain; anger management	0.35	Cognitive defusion	0.00
5	Cognitive restructuring	0.23	Practicing mindfulness	0.37
6	Stress management	0.37	Reaching acceptance	0.02
7	Time-based activity pacing	0.45	Making a commitment to action	0.05
8	Pleasant activity scheduling	0.45	Facing obstacles	0.00
9	Sleep hygiene	-	Living beyond your pain	-
10	Relapse prevention and flare-up planning; termination	-	Termination	-

**Table 2:** Components with eigenvalues exceeding Kaiser’s criterion.

Component Number	Actual Eigenvalue from PCA	Criterion Value from Parallel Analysis	Decision
1	8.088	1.943	Accept
2	1.611	1.769	Reject
3	1.486	1.635	Reject
4	1.241	1.519	Reject
5	1.097	1.406	Reject
